# Mediterranean Diet and Atrial Fibrillation: Lessons Learned from the AFHRI Case–Control Study

**DOI:** 10.3390/nu14173615

**Published:** 2022-09-01

**Authors:** Felix Alexander Neumann, Bettina Jagemann, Nataliya Makarova, Christin Susanna Börschel, Ghazal Aarabi, Friederike Gutmann, Renate B. Schnabel, Birgit-Christiane Zyriax

**Affiliations:** 1 Preventive Medicine and Nutrition, Midwifery Science - Health Services Research and Prevention, Institute for Health Services Research in Dermatology and Nursing (IVDP), University Medical Center Hamburg-Eppendorf (UKE), 20246 Hamburg, Germany; 2 German Center for Cardiovascular Research (DZHK), Partner site Hamburg/Kiel/Lübeck, 20246 Hamburg, Germany; 3 Department of Cardiology, University Heart & Vascular Center Hamburg-Eppendorf (UHZ), University Medical Center Hamburg-Eppendorf (UKE), 20246 Hamburg, Germany; 4 Department of Periodontics, Preventive and Restorative Dentistry, University Medical Center Hamburg-Eppendorf (UKE), 20246 Hamburg, Germany; 5 Max-Delbrück-Center for Molecular Medicine in the Helmholtz Association (MDC), 13125 Berlin, Germany; 6 Charité—Universitätsmedizin Berlin, Charitéplatz 1, Corporate Member of Freie Universität Berlin and Humboldt-Universität zu Berlin, 10117 Berlin, Germany; 7 Experimental and Clinical Research Center (ECRC), a Cooperation between the Max-Delbrück-Center for Molecular Medicine in the Helmholtz Association and the Charité—Universitätsmedizin Berlin, 13125 Berlin, Germany; 8 Berlin Institute of Health (BIH) at Charité—Metabolomics Platform, 13125 Berlin, Germany

**Keywords:** mediterranean diet, MEDAS, German dietary guidelines, Healthy Eating Index, dietary patterns, atrial fibrillation, cardiovascular disease, NT-proBNP

## Abstract

A relationship between lifestyle, diet, and atrial fibrillation (AF) remains unclear. Except for alcohol consumption, AF guidelines do not differentiate specific advice for this rhythm disorder. The aim of this study was to investigate the association between adherence to healthy dietary patterns and the presence of AF, among 104 low risk participants from the 1:1 matched case–control AFHRI (Atrial Fibrillation in High-Risk Individuals) study. Dietary data were obtained using a three-day food record. Adapted German versions of the validated 14-item Mediterranean Diet Adherence Screener (MEDAS) and the validated eight-item Healthy Eating Index (HEI) from the Epic Study served as the basis for data derivation. The median age of the study participants was 63.0 years, 73.1% were men. In multivariable adjusted binary logistic regression analyses, we found inverse associations between both dietary indices (MEDAS: Median = 3, HEI: Median = 54.9) and the presence of AF (odds ratio for MEDAS: 0.65, 95% confidence interval (CI): 0.47–0.91, odds ratio for HEI: 0.60, 95% CI 0.39–0.95). Further clinical studies are needed to confirm the extent to which high quality dietary patterns such as a Mediterranean diet influence the onset and natural history of AF, in order to provide dietary counselling.

## 1. Introduction

Atrial fibrillation (AF) is the most common cardiac arrhythmia, with increasing incidence [1,2,3] and prevalence over the past 30 years [4]. It is associated with an increased risk of myocardial infarction and stroke, which can be predicted by elevated levels of N-terminal pro-b-natriuretic peptide (NT-proBNP) [5,6,7] compared to the general population. Several factors are considered to contribute to its development, including age, gender, hypertension, type 2 diabetes mellitus, and modifiable lifestyle-related factors such as obesity [8,9,10,11,12,13]. Along with this, ≥10% weight reduction was found to be associated with the reduction of AF recurrences among obese patients [14] and reduced risk for de novo AF [15].

Healthy diets based on the Mediterranean diet or the Healthy Eating Index (HEI), have been shown to have a preventive effect in the context of cardiovascular disease (CVD) onset [16,17,18]. Accordingly, the prevention guidelines for CVD provide dietary recommendations [19]. However, except for the recommendation to reduce alcohol consumption, as alcohol was positively associated with AF risk [20], there have been no specific recommendations on nutrition in the AF guidelines to date [21]. This could result from the fact that there is little evidence to support a particular dietary approach [22]. While plant-based diets have been shown to reduce the likelihood of many traditional AF-related risk factors, including hypertension, hyperthyroidism, obesity, type 2 diabetes mellitus, inflammation, or and subclinical atherosclerosis [23], initial studies have suggested that a Mediterranean diet can reduce the risk of AF onset and contribute to reversing AF in patients [17,24]. To isolate nutritional aspects from confounding by co-existing risk factors and prevalent CVD, it is advantageous to investigate patients with low prevalence of classical risk factors. 

With regard to individual dietary components, coffee consumption has shown to be associated with lower AF incidence rates [25,26,27]. Moreover, for fish consumption or n-3 polyunsaturated fatty acid intake and incidence of AF, no association was found [28,29]. To the best of our knowledge, no disease-specific indices have been validated to assess the quality of diet in patients with AF. Practical instruments for assessing dietary patterns include the Mediterranean Diet Adherence Screener (MEDAS) and the HEI. Based on randomised controlled trials, a Mediterranean diet is recommended in European and international guidelines as a “healthy heart” diet [30,31,32,33]. Adherence can be assessed by the 14-item MEDAS [34]. The validated 8-item HEI is a European model based on dietary habits according to recommended eating patterns and is associated with a reduced risk of CVD [35,36,37].

The present matched case–control study therefore aimed (a) to explore group differences in dietary patterns of AF patients compared with controls, (b) to explore whether dietary patterns correlated with known AF-related risk factors, (c) to investigate the association of adherence to healthy dietary patterns measured by indices scores (MEDAS or HEI) with the presence of AF, and additionally (d) to investigate whether such an association was related to an AF-related blood biomarker.

## 2. Materials and Methods

### 2.1. Design, Setting and Participants

Atrial Fibrillation in High-Risk Individuals (AFHRI) is a prospective, monocentric clinical cohort study to improve the prediction of personal risk for AF, conducted at the University Heart and Vascular Center, Hamburg. Between October 2019 and March 2020, we recruited a sub-sample of AF patients from the AFHRI cohort (AFHRI-C). It was ensured that the selected AF patients had a limited risk-factor burden, in particular no prevalent cardiovascular disease, thyroid dysfunction, or cancer. Because the development of CVD often interacts with metabolic risk factors including hypertension, obesity, hyperlipidaemia, and type 2 diabetes mellitus [19], we expected that the sub-sample of the study population with limited risk-factor burden would allow insight into isolated influences of lifestyle and diet on risk of arterial prolapse. The sub-sample was matched to controls based on age, gender, and risk factor (matching criteria), selected from the Hamburg City Health Study (HCHS), a prospective, long-term, population-based cohort study, conducted at the University Medical Center Hamburg-Eppendorf [38]. In total, 153 participants were enrolled—68 AF patients and 85 matched controls. The study was approved by the local ethics committee and conducted in accordance with the Declaration of Helsinki, and written informed consent was obtained.

### 2.2. Variables, Measurements and Processes

Questionnaire data and peripheral venous blood were acquired on the day of enrolment. The participants’ characteristics were collected through a questionnaire administered by a healthcare professional, and from patient files. Variables of the present analysis included gender, age, height, prevalent diseases, medication including vitamin K antagonists, CVD risk factors such as alcohol consumption, current and former smoking status, physical activity, dietary intake, dietary changes within the year prior to examination, as well as income (Table S1). Income was deliberately chosen over other socio-economic variables since it is associated with education as well as improved food choice and quality, hence was more relevant to the study [39]. Quality of patients’ medical data was controlled via information from patients’ medical records. To measure physical activity, we used the validated seven-item International Physical Activity Questionnaire short form (IPAQ-SF) [40] and calculated metabolic equivalents (MET) of physical activity. Resting heart rate (beats per minute) was measured after 5 min in sitting position, with a 12-channel ECG (Schiller^®^ CARDIOVIT AT-10 plus). Blood samples were taken and NT-proBNP (pg/mL) was determined as an AF-related blood marker (Elecsys^®^ proBNP II, Roche). Patient weight was measured with a calibrated mechanical column scale (Seca 709). The body mass index (BMI) was defined as body weight in kg/body height in m^2^. Underweight was defined as BMI up to 18.5 kg/m^2^, normal weight as BMI ≥ 18.5 kg/m^2^ up to 25 kg/m^2^, overweight as BMI ≥ 25 kg/m^2^ up to 30 kg/m^2^, and obesity as BMI ≥ 30 kg/m^2^.

To assess nutrition, the participants were asked to complete a three-day dietary record before their first study participation appointment. To improve data quality, we used a standardised procedure during data collection with precise questions administered by only one study nurse. The food data was analysed in terms of energy and nutrient intake and the adherence to the dietary patterns of the Mediterranean Diet Adherence Screener (MEDAS), and the Healthy Eating Index (HEI) was derived from patients’ food intake (Tables S2 and S3). The 14-item MEDAS includes the frequency of food consumption (olive oil, vegetables, fruits, red meat, animal fats, carbonated drinks, red wine, fish/seafood, legumes, nuts, commercial foods, and traditional Mediterranean dishes with tomato sauce) as well as the preferred cooking fat and meat consumed [34]. Each item was scored zero or one depending on whether the item-specific criteria were met, resulting in a scores between 0 and 14. Since different versions of the HEI are available, we decided to use the edition validated in German, which appeared to be suitable for the high-quality nutritional assessment in Germany [41]. Based on food protocols, this German version of the HEI scores five food groups from 0 to 10 (cereal and potato; dairy; meat, sausage, fish, and egg; fat or oil; sweets and foods high in fat) and three food groups from 0–20 (vegetables; fruits; beverages), allowing total scores between 0 and 110 points. Information on how food portions were converted into score points is included in the Supplementary Data. For the evaluation of the nutrition data, the Software Ebispro 2011 (Stuttgart, Germany) was used, which includes 15,000 foods (food items) based on the German food composition database BLS version 3.01 (Karlsruhe, Germany).

### 2.3. Data Handling

The variables were tested using Shapiro–Wilk tests for normal distribution and visualisations such as scatter plots and box plots for outliers. MET and NT-proBNP showed strong positive skew in their distribution and therefore variables were transformed by applying common logarithms to base 10 as appropriate [42]. After checking whether missing values were distributed randomly, using Little’s MCAR Test (chi^2^ (67) = 72.91, *p* = 0.290), we performed a multiple data imputation with five imputations [43] and aggregated these into a pooled value using the Bar procedure [44]. Missing values were imputed for income (15), resting heart rate (12), NT-proBNP (8), and weight (2). To explore the impact of the imputation on our results, we ran our models several times excluding once each variable with imputed data. However, we were not able to detect differences of relevance. Further information about the imputation including pre-post-comparison of imputed variables, variables used for multiple imputation, and complete case result tabulations are presented in Table S6. 

### 2.4. Participants and Matching

**Figure 1:**
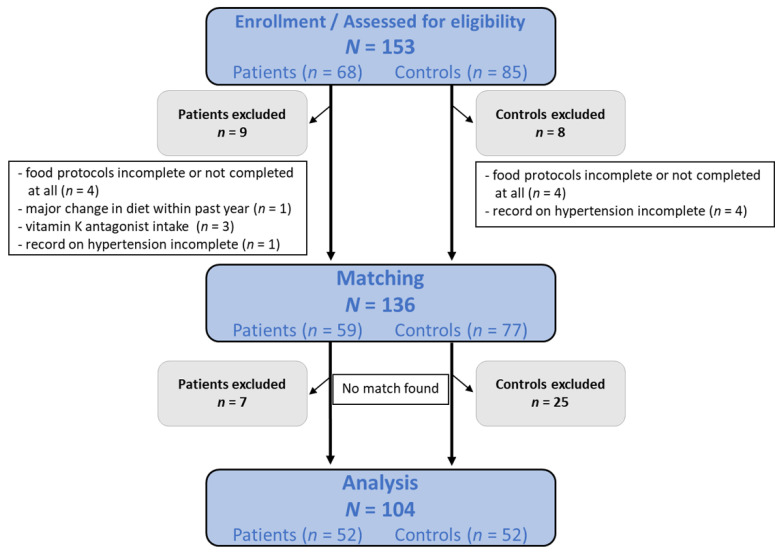
Flow chart of the study population selection.

Before matching patients and controls, 17 participants were excluded. Reasons for study exclusion were noncompleted or poorly completed dietary protocols, major dietary changes within the past year, incomplete records on pre-existing arterial hypertension, and intake of vitamin K antagonists. Matching was based on gender, pre-existing arterial hypertension, and a maximum age difference of eight years. For the statistical analysis, 104 participants remained, 52 patients and 52 controls (Figure 1). In a pre-post comparison for matching, no significant differences were found in the descriptive statistics of key variables used for modelling (Table S7).

### 2.5. Statistical Analysis

Categorical variables of participant characteristics are presented by their absolute and relative frequencies, and Pearson’s chi-square and Fisher’s exact tests were used to determine subgroup comparisons. Shapiro–Wilk tests indicated that most of the continuous variables significantly varied from normal distribution. Therefore, continuous variables are reported as the median and the first and third quartiles. Non-parametric tests for group comparisons (Mann–Whitney U tests), and correlations of variables (Spearman’s rho) were applied. The size of each significant correlation coefficient was interpreted according to rule of thumb (negligible: R < 0.3, low: 0.3 < R < 0.5, moderate: 0.5 < R < 0.7, high: 0.7 < R < 0.9, very high: 0.9 < R < 1.0) [45]. To analyse the association of the nutrition scores with the presence of atrial fibrillation, we performed binary logistic regression analysis in two different models for MEDAS and HEI. Independent variables which correlated (R > 0.3) with MEDAS and HEI or with each other were excluded from both models to avoid effects of multicollinearity. The crude model equals a Pearson correlation (unadjusted). Model 1 was adjusted for income, resting heart rate, log MET, energy intake, and dietary changes over the past year. Model 2 for both nutrition scores was adjusted for the variables of Model 1 and additionally for log NT-proBNP. All possible confounders were tested for significance within the model. To make comparable the results of the binary logistic regressions for the two dietary patterns, the maximum HEI score was scaled down to the maximum MEDAS score, from 110 to 14. Thus, a one-point increase in the model was equivalent to a one-point increase in MEDAS and a $\frac{110}{14}\approx$ 7.86-point increase in the HEI. Matching variables (gender [±0], age [±8 years], hypertension [±0]) as well as closely related variables (e.g., smoking status) were not taken into account for modelling, as the matching procedure aimed to eliminate the effects of these variables. Wald tests were applied to determine whether odds ratios (OR) differed from 1. Results of the regressions are presented as ORs and their 95% lower and upper confidence intervals (CI). Nagelkerke’s R^2^ was used to describe the regression model’s fit. The significance level was set to a < 0.05. All statistical tests were computed using SPSS Statistics (version 26.0; IBM Corp., Armonk, NY, USA) and Microsoft Excel 2013 (Microsoft, Redmond, WA, USA).

## 3. Results

### 3.1. Characteristics of Study Participants

We included N = 104 participants in this analysis; 52 patients with AF but limited risk-factor burden and 52 controls. Based on the matching procedure, the groups were equal in their proportion of men (73.1%). Furthermore, there were no significant differences in age, income, prevalence of diseases such as type 2 diabetes mellitus, dyslipidaemia, and peripheral arterial disease, or for reported partial dietary changes within the past 12 months. Clinical parameters such as body mass index (z = 3.32, *p* = 0.001), log NT-proBNP (z = 5.61, *p* < 0.001), and resting heart rate (z = 2.33, *p* = 0.020) were significantly lower for the control group compared to the patient group. In contrast, patients had significantly lower scores on both nutritional scales, MEDAS (z = 2.47, *p* = 0.014) and HEI (z = 2.93, *p* = 0.003). More detailed information on study participants is provided in Table 1.

### 3.2. Group Differences in Dietary Patterns

**Figure 2:**
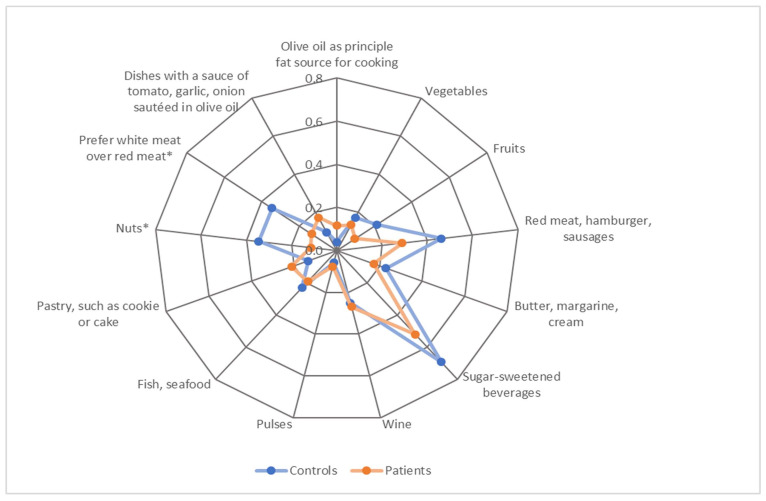
Dietary patterns of patients and controls according to average scores achieved for items in the Mediterranean Diet Adherence Screener (MEDAS). Note. The category “Olive oil, rapeseed oil” was excluded as no participant scored. Scores ranged between 0–1 point. * *p* < 0.05. Detailed numerical results are presented in Table S4.

**Figure 3:**
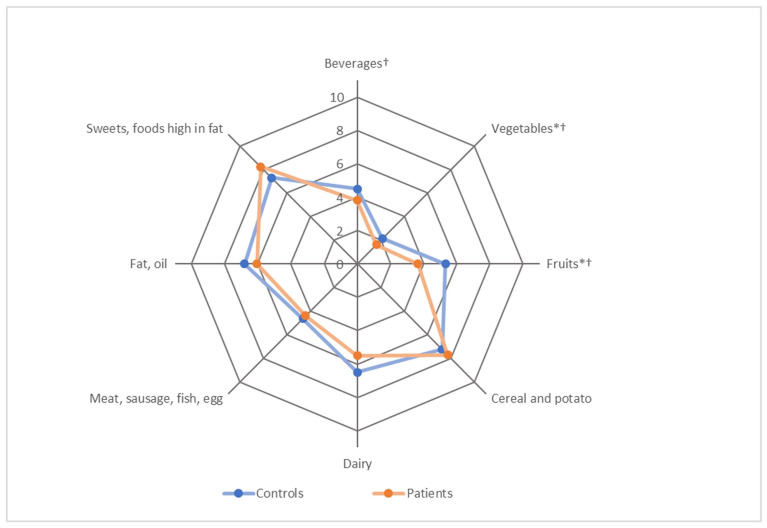
Dietary patterns of patients and controls according to average scores achieved for the items of the Healthy Eating Index (HEI). Note. Scores ranged between 0–10 or 0–20 points depending on the item. ^†^ maximum points downscaled from 20 to 10 points. * *p* < 0.05. Detailed numerical results are presented in Table S5.

For MEDAS, significant differences were found in two out of fourteen food groups. The control group scored significantly higher for the food group nuts (z = 7.80, *p* = 0.010) and for the preference of white meat over red meat (z = 6.37, *p* = 0.021), compared with AF patients. No study participant consumed four or more servings of olive or rapeseed oil per day. The adherence to MEDAS of the diets was low overall, and the median MEDAS score was three out of fourteen points. For HEI, statistical analysis revealed that the control group scored significantly higher in the vegetable (z = 2.66, *p* = 0.008) and fruit (z = 2.70, *p* = 0.007) food groups. Similar results applied for beverages (z = 1.79, *p* = 0.074) and dairy (z = 1.84, *p* = 0.066), for which the *p*-value was bordering significance. The control group scored significantly higher for both MEDAS (z = 2.47, *p* = 0.014) and HEI (z = 2.93, *p* = 0.003). The dietary patterns of patients and controls according to MEDAS and HEI are shown in Figure 2 and Figure 3.

### 3.3. Correlation Analysis

MEDAS and HEI showed a low positive correlation with each other (r = 0.322, *p* = 0.001), and each correlated negatively with the presence of AF (MEDAS: r = −0.243, *p* = 0.013; HEI: r = −0.289, *p* = 0.003), BMI (MEDAS: r = −0.300, *p* = 0.002; HEI: r = −0.394, *p* < 0.001), and resting heart rate (MEDAS: r = −0.185, *p* = 0.060; HEI: r = −0.226, *p* = 0.021). HEI also correlated positively with age (r = 0.217, *p* = 0.027) and total energy intake (r = 0.198, *p* = 0.044). However, most of these effects were negligible for the model calculation. An overview of all correlations can be found in Table 2.

### 3.4. Association of MEDAS and HEI with the Presence of AF

**Figure 4:**
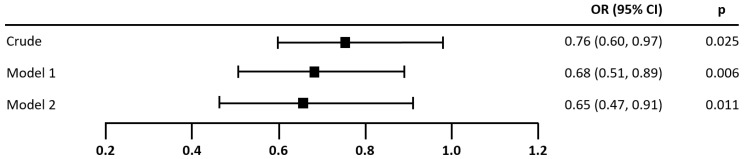
Association of MEDAS score with the presence of atrial fibrillation. Note. *N* = 104. Odds ratios (OR) for the presence of atrial fibrillation per one point increase in MEDAS. Model 1 was adjusted for income, resting heart rate, log MET, energy intake, and diet change in the past year. Model 2 was additionally adjusted for log NT-proBNP. The odds ratio for log NT-proBNP in Model 2 was significant: OR (95% CI) = 19.43 (7.41–183.27), *p* < 0.001. Wellness of fit, correct predictions, and overall model-fit statistics—Crude: Nagelkerke’s R^2^ = 0.067, 61.54%, Chi^2^ = 5.36, *p* = 0.021; Model 1: Nagelkerke’s R^2^ = 0.204, 64.42%, Chi^2^ = 17.25, *p* = 0.016; Model 2: Nagelkerke’s R^2^ = 0.491, 79.81%, Chi^2^ = 47.82, *p* < 0.001.

**Figure 5:**
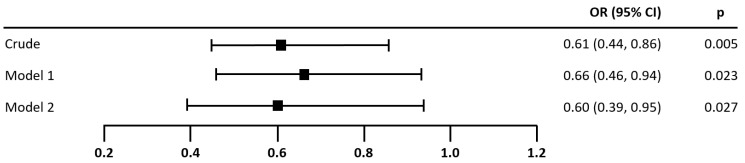
Association of HEI score with the presence of atrial fibrillation. Note. *N* = 104. Odds ratios (OR) for the presence of atrial fibrillation per 7.86 points increase in HEI. The maximum score of the HEI was scaled down from 110 to 14 for the logistic regression, in order to make the results comparable to MEDAS. Model 1 was adjusted for income, resting heart rate, log MET, energy intake, and diet change in the past year. Model 2 was additionally adjusted for log NT-proBNP. The odds ratio for log NT-proBNP in Model 2 was significant: OR (95% CI) = 36.71 (7.74–174.17), *p* < 0.001. Wellness of fit, correct predictions, and overall model-fit statistics—Crude: Nagelkerke’s R^2^ = 0.112, 61.54%, Chi^2^ = 9.15, *p* = 0.002, Model 1: Nagelkerke’s R^2^ = 0.173, 63.46%, Chi^2^ = 14.49, *p* = 0.043, Model 2: Nagelkerke’s R^2^ = 0.476, 79.81%, Chi^2^ = 45.92, *p* < 0.001.

The results of the logistic regressions in Figure 4 and Figure 5 demonstrate that MEDAS (OR = 0.76, 95% CI 0.60–0.97, *p* = 0.025) and HEI (OR = 0.61, 95% CI 0.44–0.86, *p* = 0.005) were both significantly associated with the presence of AF. This association remained significant in Model 1 after adjustment for income, resting heart rate, log MET, energy intake, and dietary changes over the past year (MEDAS: OR = 0.68, 95% CI 0.51–0.89, *p* = 0.006; HEI: OR = 0.66, 95% CI 0.46–0.94, *p* = 0.023), and for Model 2 after additional adjustment for log NT-proBNP (MEDAS: OR = 0.65, 95% CI 0.47–0.91, *p* = 0.011; HEI: OR = 0.60, 95% CI 0.39–0.95, *p* = 0.027). Model 2 is to be preferred over Model 1, as it included an additional important independent variable: log NT-proBNP. According to the likelihood ratio test statistic, Model 2 was superior to Model 1 in terms of overall model fit. The percentage of correct predictions increased by 15.39% (MEDAS) and 16.35% (HEI), Nagelkerke’s R^2^ value more than doubled (MEDAS: R^2^ = 0.491; HEI: R^2^ = 0.476), and the coefficient for log NT-proBNP was statistically significant at the 0.001 level.

## 4. Discussion

To our knowledge, this matched case–control study among AF patients with limited risk-factor burden was the first to account for potential confounders before investigating dietary patterns and their relationship to the presence of AF and known AF risk factors. Our findings indicate that (a) the dietary patterns of patients with prevalent AF were poorer than those of age-, gender- and risk factor-matched control individuals, both in terms of adherence to individual foods such as nuts, vegetables and fruits, and to MEDAS and HEI overall; (b) MEDAS and HEI both correlated with AF-related risk factors such as BMI and resting heart rate in patients with AF; (c) the adherence to healthy dietary patterns measured by both MEDAS and HEI was independently negatively associated with the presence of AF, even after adjustments; and (d) inclusion of NT-proBNP as a typical blood biomarker for AF remains significant.

Lifestyle and nutrition have already been shown to be influencing factors for a multitude of cardiovascular conditions [19]. Additionally, the incidence and prevalence of AF is increasing in ageing societies [4]. According to the guidelines of the European Society of Cardiology, in addition to age, about 50% of the known risk factors for AF are associated with lifestyle, leading to diseases such as diabetes, dyslipidaemia, hypertension, obesity, and early coronary heart diseases [21]. Consequently, lifestyle changes are recommended, including reduction of weight, lower alcohol consumption, and smoking cessation, as well as increased regular exercise. Although individual aspects of nutrition have been examined in relation to AF, e.g., caffeine intake [25,26,27] or fish consumption [28], little evidence overall exists to complete the complex nutritional picture. No specific dietary recommendations can be found in guidelines for AF. Initial studies have indicated that the Mediterranean diet [17,24] or a plant-based diet [23] may be beneficial in preventing or even reversing the development of AF, so we analysed associations of dietary patterns as measured by MEDAS, which incorporates several of the above factors. The MEDAS evaluation not only revealed that the group of AF patients performed significantly less well compared to the controls, but also an independent inverse relationship between adherence to healthy dietary patterns, assessed by MEDAS, and the presence of AF. Differences for single items were found in the categories “nuts” and “preference for white meat over red meat”, where the AF patients had significantly less beneficial intake. In comparison to previous analysis carried out by HCHS, adherence to MEDAS was slightly lower in our study sample (M = 2.88 vs. M = 4.54 out of 14) which can be attributed to differences in the survey instruments as well as data derivation [46]. Overall, both groups in our study showed low adherence to MEDAS, suggesting that the Mediterranean diet is only reflected to a small degree in the dietary patterns of the German population [47]. However, since a rather low adherence to the Mediterranean diet was to be expected, we analysed an additional nutrition construct, the HEI, which had been developed in an international setting for CVD and adapted according to German dietary guidelines. The latter is reflected in our descriptive results for HEI in the control group (Median = 56.5), which were similar to the German population average found by von Rüsten et al. (M_men_ = 54.9, M_women_ = 59.8) [41]. The HEI was therefore a suitable tool for our study with German participants. Although we additionally used dietary patterns adapted to German eating habits, our AF patients still scored significantly less well compared with the controls, particularly for the food groups “fruits” and “vegetables” as well as overall (Med = 51.7). The results for HEI were similar to those for MEDAS. It was found that the odds of the presence of AF decreased by 40% (95% CI 5%–61%) with a 7.86-unit increase in HEI. Hence, the results of both dietary scores pointed in the same direction and, unsurprisingly, MEDAS and HEI showed a correlation to each other. Although the indices share some identical items, MEDAS and HEI differ fundamentally in scoring, number and composition of items, and targeted population. This is because the MEDAS has fixed cut-off values for scoring, which may allow group differences to remain undetected that are revealed in HEI. From a critical point of view, both dietary indices seem less than ideal for the study participants examined, so development of an index that provides a more accurate classification of food groups could lead to even better results. In particular, the food groups “meat, sausage, fish, egg” and “fat, oil” in the HEI require improved classification to be used in the investigation of patients with CVD and AF. An analysis of six prospective cohort studies found that a higher intake of different types of meat, but not fish, was significantly associated with a small increase in the risk of incident CVD [48]. Furthermore, in a randomised trial, butter and olive oil were associated with opposite effects on blood lipid levels [49], which are an established risk factor for CVD [19]. Based on these findings, the Alternate Healthy Eating Index [50], which showed a strong inverse association with CVD in a prospective cohort study, could be usefully applied in the study of AF. However, the low to mediocre adherence to the survey instruments used also raises the question of whether poor adherence is due to the surveys’ failures to reflect accurately the dietary habits of the German population and the degree to which the German population lacks a healthy diet in general. Regardless, our cross-sectional results indicated that MEDAS and HEI were associated with AF-related risk factors, such as BMI [51] and resting heart rate [52], and with the presence of AF in patients with limited risk-factor burden. Therefore, more in-depth investigation is required, applying a study design with higher evidence levels, such as prospective intervention studies.

### Strengths and Limitations

Certain limitations of this study should be mentioned. First, due to the small sample size, it is possible that group differences in dietary patterns remained undetected. Second, the present study followed a case–control design and thus precluded any causal interpretations. Third, our calculations were not adjusted for multiple testing, which leaves the possibility of undetected α-error. Finally, there is evidence that additional factors such as emotional distress and obstructive sleep apnoea are associated with AF [21], and these could have been considered as potential confounders. 

Our study has several strengths, in particular its methodological approach. We focussed on minimising confounding factors at all times during the study. For example, suitable participants for study inclusion were carefully selected from the cohort, one nurse exclusively conducted food questionnaires, and the collected data quality was controlled by a nutritionist. Participants who reported having changed their dietary patterns within the last year, had poorly completed dietary records, or took vitamin K antagonists were excluded from the analysis. Matching was based on age, gender and risk factors. All requirements were checked before multivariate analysis, and the model was appropriately adjusted. A special feature of our evaluation was that we extracted data for both MEDAS and HEI from the patients’ dietary records, which allowed a deeper understanding of the data and indices. 

## 5. Conclusions

In ageing populations, AF is of high public health relevance, yet there is little evidence on the importance of dietary patterns. We were able to show that adherence to the Mediterranean diet, and especially the intake of plant-based foods such as nuts, vegetables, and fruits, and also preference for white meat over red meat, was less frequently reported by AF patients. Larger clinical studies are required to confirm the extent to which high quality dietary patterns such as a Mediterranean diet are able to influence the onset of AF, in order to provide dietary recommendations for preventive nutrition counselling. Beyond this, the development of a disease-specific nutritional assessment score is desirable to offer an opportunity to enhance secondary prevention in AF, as it could be used for taking medical histories by physicians and for the empowerment of affected individuals.

## Figures and Tables

**Table 1:** Characteristics of study participants.

Food Items	Total (*N* = 104)	Patients (*n* = 52)	Controls (*n* = 52)	|Standardised Test Statistic|	*p*-Value
Sociodemographic and Clinical Data					
Male	76 (73.1%)	38 (73.1%)	38 (73.1%)	0 ^a^	1.000
Age, years	63.0 (53.8, 69.4)	63.7 (53.8, 69.6)	62.5 (53.3, 69.4)	0.23 ^c^	0.820
Age categories				2.06 ^b^	0.336
<65	58 (55.8%)	27 (51.9%)	31 (59.6%)		
65 < 75	42 (40.4%)	24 (46.2%)	18 (34.6%)		
>75	4 (3.8%)	1 (1.9%)	3 (5.8%)		
Income categories				0.35 ^a^	0.866
low (<2500€)	19 (18.3%)	9 (17.3%)	10 (19.2%)		
middle (2500€ < 5000€)	55 (52.9%)	29 (55.8%)	26 (50.0%)		
high (>5000€)	30 (28.8%)	14 (26.9%)	16 (30.8%)		
Body mass index, kg/m^2^	25.2 (23.0, 27.3)	26.3 (23.9, 28.6)	24.2 (22.2, 26.9)	3.32 ^c^	**0.001**
Body mass index categories				9.48 ^b^	**0.011**
Underweight	1 (1.0%)	0 (0%)	1 (1.9%)		
Normal weight	49 (47.1%)	18 (34.6%)	31 (59.6%)		
Overweight	47 (45.2%)	28 (53.8%)	19 (36.5%)		
Obesity	7 (6.7%)	6 (11.5%)	1 (1.9%)		
NT-proBNP, pg/ml	95.25 (50.5, 225.6)	210.7 (96.1, 379.1)	64.7 (40.4, 93.8)	5.61 ^c^	**<0.001**
Resting heart rate, bpm	61.0 (56.0, 68.3)	64.0 (58.0, 71.3)	59.0 (54.0, 66.5)	2.33 ^c^	**0.020**
Prevalent diseases					
Type 2 diabetes mellitus	2 (1.9%)	0 (0%)	2 (3.8%)	2.81 ^b^	0.495
Arterial hypertension	14 (13.5%)	7 (13.5%)	7 (13.5%)	0 ^a^	1.000
Dyslipidaemia	19 (18.3%)	8 (15.4%)	11 (21.2%)	0.58 ^a^	0.613
Blood-thinning medications ^d^	0 (0%)	0 (0%)	0 (0%)	0 ^b^	1.000
Anticoagulants ^e^	18 (17.3%)	18 (34.6%)	0 (0%)	19.20 ^a^	**<0.001**
Lifestyle factors					
MEDAS, points	3 (1.25, 4)	2 (1, 4)	3 (2, 5)	2.47 ^c^	**0.014**
Healthy Eating Index, points	54.9 (47.2, 60.6)	51.7 (45.1, 57.5)	56.5 (51.0, 63.8)	2.93 ^c^	**0.003**
Healthy Eating Index, categories				8.85 ^a^	**0.011**
poor (≤40 pts.)	11 (10.6%)	8 (15.4%)	3 (5.8%)		
improvable (>40–64 pts.)	77 (74.0%)	41 (78.8%)	36 (69.2%)		
good (>64 pts.)	16 (15.4%)	3 (5.8%)	14 (25.0%)		
Diet change past 12 months				0.54 ^a^	0.626
no	83 (79.8%)	40 (76.9%)	43 (82.7%)		
yes, partially	12 (23.1%)	12 (23.1%)	9 (17.3%)		
Energy intake, kcal/day	2179.5 (1901.7, 2484.2)	2137.4 (1885.3, 2417.0)	2206.3 (1918.9, 2544.8)	0.54 ^c^	0.589
Physical activity, MET-h/day	2162.0 (1350.4, 3981.8)	2857.0 (1360.1, 5716.5)	2016.8 (1350.4, 3154.5)	1.69 ^c^	0.091
Alcohol consumption	84 (80.8%)	42 (80.8%)	42 (80.8%)	0 ^a^	1.000
Current and former smoking	67 (64.4%)	37 (71.2%)	30 (57.7%)	2.06 ^a^	0.219

Note. Data presented as percentages for categorical variables and as median (1st quartile, 3rd quartile) for continuous variables. ^a^ Pearson’s chi square test. ^b^ Fisher’s exact test. ^c^ Mann–Whitney U test. ^d^ Phenprocuomon, Warfarin, and further. ^e^ Dabigantran, Apixaban, Rivaroxaban, and further. Multiple data imputation with five imputations [43] was performed to fill in for missing values of income (*n* = 15), resting heart rate (*n* = 12), NT-proBNP (*n* = 8), and weight (*n* = 2), based on *N* = 153. Significant correlations (*p* < 0.05) marked in bold.

**Table 2:** Correlations of Mediterranean Diet Adherence Screener (MEDAS) and Healthy Eating Index (HEI) with independent variables.

		MEDAS		HEI
	R	*p*	R	*p*
Presence of AF	−0.243	**0.013**	−0.289	**0.003**
MEDAS	-		0.322	**0.001**
HEI	0.322	**0.001**	-	
Age	0.092	0.352	0.217	**0.027**
Income	0.069	0.488	−0.008	0.939
BMI	−0.300	**0.002**	−0.394	**<0.001**
Log NT-proBNP	−0.090	0.364	−0.129	0.190
Resting heart rate	−0.185	0.060	−0.226	**0.021**
Diet change past year	0.215	**0.028**	−0.020	0.838
Energy intake	−0.050	0.614	0.198	**0.044**
Log MET	0.120	0.225	−0.161	0.102

Note. Spearman correlation coefficients of MEDAS and HEI with independent variables (*n* = 104). AF, atrial fibrillation; BMI, body mass index; MET, metabolic equivalents. Significant correlations (*p* < 0.05) marked in bold.

## Data Availability

The data analysed during the current study are not publicly available due to the German National Data Protection Regulation. They are available on reasonable request from the corresponding author.

## Supplementary Materials

The following are available online at https://www.mdpi.com/article/10.3390/nu14173615/s1, Table S1: Listing of study variables sorted by assessment instrument; Table S2: Coding for the translation of food intake to points in the Mediterranean Diet Adherence Screener; Table S3: Coding for the translation of food intake to points in the Healthy Eating Index; Table S4: Group differences in the food items of the Mediterranean Diet Adherence Screener; Table S5: Group differences in the food items of the Healthy Eating Index; Table S6: Case result tabulations for each step of the multiple data imputation; Table S7: Comparison of main variables pre- and post-case-control matching.
