# Supplementary material for: Mediterranean Diet and Atrial Fibrillation: Lessons Learned from the AFHRI Case–Control Study
Source: Nutrients. 2022 Sep 1;14(17):3615. doi: 10.3390/nu14173615 (PMC9459737; doi:10.3390/nu14173615)
Supplement: Supplementary file 1 [file nutrients-14-03615-s001.zip › nutrients-1839276-supplementary.pdf]

Table S1. Listing of study variables sorted by assessment instrument

Assessment instruments	Study variables
Questionnaire (self-administered)	Gender, age, income, dietary changes within a year prior to examination, alcohol consumption, current and former smoking status
Questionnaire (self-administered) and patient's file	Prevalent diseases (type 2 diabetes mellitus, arterial hypertension, dyslipidaemia)
Patient's file	Height, medication (blood-thinning medications, anticoagulants)
Calibrated mechanical column scale (Seca 709)	Weight
Blood sampling	N-terminal pro-b-natriuretic peptide
12-channel electrocardiogram (Schiller® CARDIOVIT AT-10 plus)	Resting-heart rate
Questionnaire (IPAQ-SF)	Physical activity
Three-day dietary record	Dietary intake (energy intake, Mediterranean Diet Adherence Screener (MEDAS), Healthy Eating Index (HEI))

Table S2. Coding for the translation of food intake to points in the Mediterranean Diet Adherence Screener (MEDAS)

No.	Foods and frequency of consumption	Criteria for 1 point*
1	Do you use olive oil/rapeseed oil as main culinary fat?	Yes
2	How much olive oil do you consume in a given day (including oil used for frying, salads, out of house meals, etc.)?	4 or more tablespoons
3	How many vegetable servings do you consume per day? (1 serving = 200g - consider side dishes as 1/2 serving)	2 or more (at least 1 portion raw or as salad)
4	How many fruit units (including natural fruit juices) do you consume per day?	3 or more
5	How many servings of red meat, hamburger, or meat products (ham, sausage, etc.) do you consume per day? (1 serving = 100-150 g)	Less than 1
6	How many servings of butter, margarine, or cream do you consume per day? (1 serving = 12 g)	Less than 1
7	How many sweet/carbonated beverages do you drink per day?	Less than 1
8	How much wine do you drink per week?	7 or more glasses
9	How many servings of legumes do you consume per week? (1 serving = 150 g)	3 or more
10	How many servings of fish or shellfish do you consume per week? (1 serving: 100-150 g fish, or 4-5 units or 200 g shellfish)	3 or more
11	How many times per week do you consume commercial sweets or pastries (not homemade), such as cakes, cookies, biscuits, or custard?	Less than 3
12	How many servings of nuts (including peanuts) do you consume per week? (1 serving = 30 g)	3 or more
13	Do you preferentially consume chicken, turkey or rabbit meat instead of veal, pork, hamburger or sausage?	Yes
14	How many times per week do you consume vegetables, pasta, rice, or other dishes seasoned with sofrito (sauce made with tomato and onion, leek, or garlic, simmered with olive oil)?	2 or more

Note. *0 points if these criteria are not met.

Modified from: Estruch R, Ros E, Salas-Salvadó J, et al. Primary prevention of cardiovascular disease with a Mediterranean diet. N Engl J Med 2013;368:1279-90. DOI: 10.1056/NEJMoa1200303 [20]

Table S3. Coding for the translation of food intake to points in the Healthy Eating Index (HEI)

Food group	Food subgroup	Serving size (f/m)	Servings per day
(1) Beverages	(1) Beverages	280 ml	6
	Juices	100 ml	
(2) Vegetables	(2) Vegetables	140 g	3
	(3) Vegetable juice[a]	100 ml	
(3) Fruit	(4) Fruit	125 g	2
	(5) Fruit juice[a]	100 ml	
(4) Cereals, cereal products,	(6) Bread, cereal flakes	70/85 g	4
potatoes	(7) Side dishes (potatoes, pasta, rice)	200/250 g	
(5) Milk and milk products	(8) Milk	250 ml	3
	(9) Yoghurt	150 g	
	(10) Cheese	30 g	
(6) Meat, sausage, fish, egg	(11) Meat	200 g	1
	(12) Sausage	30 g	
	(13) Fish	150-200 g	
	(14) Egg	2-3 pieces	
(7) Fats and oils	(15) Fats	18/20 g	2
	(16) Oils	18/20 g	
(8) Sweets, fatty snacks	(17) Sweets, snacks	220/270 kcal	1

Note. [a]Only the first portion of juice, additional portions of juice were counted in the "beverages" group

Table S4. Group differences in the food items of the Mediterranean Diet Adherence Screener (MEDAS)

Food items	Total (N = 104)	Patients (n = 52)	Controls (n = 52)	ǀ Standardised test statistic ǀ	p-value
Olive Oil/Rapeseed Oil as main fat source for cooking	8 (7.7%)	6 (11.5%)	2 (3.8%)	2.17	0.269
Olive Oil/Rapeseed Oil	0 (0%)	0 (0%)	0 (0%)	-	-[a]
Vegetables	16 (15.4%)	7 (13.5%)	9 (17.3%)	0.30	0.787
Fruit	16 (15.4%)	5 (9.6%)	11 (21.2%)	2.66	0.173
Red meat, hamburger, sausages	39 (37.5%)	15 (28.8%)	24 (46.2%)	3.32	0.105
Butter, margarine, cream	21 (20.2%)	9 (17.3%)	12 (23.1%)	0,54	0.626
Sugar-sweetened beverages	63 (60.6%)	27 (51.9%)	36 (69.2%)	3.26	0.108
Wine	27 (26.0%)	14 (26.9%)	13 (25.0%)	0.05	1.000
Pulses	7 (6.7%)	4 (7.7%)	3 (5.8%)	0.15	1.000
Fish, seafood	22 (21.2%)	10 (19.2%)	12 (23.1%)	0.71	0.399
Pastry, such as cookie or cake	18 (17.3%)	11 (21.2%)	7 (13.5%)	1.08	0.438
Nuts	24 (23.1%)	6 (11.5%)	18 (34.6%)	**7.80**	**0.010**
Prefer white meat over red meat	25 (24.0%)	7 (13.5%)	18 (34.6%)	**6.37**	**0.021**
Dishes with a sauce of tomato, garlic, onion sautéed in olive oil	14 (13.5%)	9 (17.3%)	5 (9.6%)	1.32	0.390

Note. Data presented as amount (percent) and test statistics of Pearson's Chi Square. [a]No statistics could be computed for Olive Oil/Rapeseed Oil.

Table S5. Group differences in the food items of the Healthy Eating Index (HEI)

Food items	Total (N = 104)	Patients (n = 52)	Controls (n = 52)	ǀ Standardised test statistic ǀ	P-value
Beverages	8.45 (6.20, 11.98)	7.60 (5.73, 9.95)	9.00 (6.63, 12.70)	1.79	0.074
Vegetables	3.80 (2.30, 6.23)	3.25 (1.93, 5.10)	4.30 (3.13, 6.90)	**2.66**	**0.008**
Fruit	8.45 (4.53, 12.58)	7.30 (4.13, 10.80)	10.65 (5.83, 15.98)	**2.70**	**0.007**
Cereal and potato	7.35 (5.80, 8.40)	7.70 (5.83, 8.58)	7.25 (5.73, 8.28)	0.71	0.480
Dairy	5.95 (4.20, 8.28)	5.50 (3.38, 7.85)	6.50 (4.70, 8.58)	1.84	0.066
Meat, sausage, fish, egg	4.50 (3.20, 5.88)	4.40 (3.30, 6.88)	4.65 (3.03, 5.78)	0.64	0.520
Fat, oil	6.55 (5.10, 7.98)	6.05 (5.13, 8.00)	6.80 (5.03, 7.85)	0.32	0.752
Sweets, foods high in fat	7.50 (5.23, 10.00)	8.25 (4.93, 10.00)	7.30 (5.23, 9.83)	0.90	0.368
Score	54.85 (47.23, 60.58)	51.70 (45.13, 57.45)	56.45 (51.03, 63.83)	2.93	0.003

Note. Data presented as median (1st quartile, 3rd quartile). Mann-Whitney U tests.

Table S6. Case result tabulations for each step of the multiple data imputation

Imputation Number			Income	Body weight	Resting heart rate	NT-proBNP
Original data	N	Valid	121	141	129	132
		Missing	22	2	14	11
	Mean		8.36	78.33	62.48	165.97
	Median		9.00	77.00	61.00	81.90
	Std. Deviation		2.59	13.77	11.26	344.77
	Minimum		2	51	37	14.60
	Maximum		11	125	114	3545.20
1	N	Valid	143	143	143	143
		Missing	0	0	0	0
	Mean		8.18	78.29	62.55	187.71
	Median		9.00	77.00	61.00	90.40
	Std. Deviation		2.55	13.68	11.41	360.68
	Minimum		2	51	37	14.60
	Maximum		11	125	114	3545.20
2	N	Valid	143	143	143	143
		Missing	0	0	0	0
	Mean		8.33	78.05	62.52	185.85
	Median		9.00	77.00	61.00	91.00
	Std. Deviation		2.55	13.88	11.16	347.58
	Minimum		2	51	37	14.60
	Maximum		11	125	114	3545.20
3	N	Valid	143	143	143	143
		Missing	0	0	0	0
	Mean		8.28	78.34	62.83	184.29
	Median		9.00	77.00	61.00	89.40
	Std. Deviation		2.54	13.70	11.11	348.43
	Minimum		2	51	37	14.60
	Maximum		11	125	114	3545.20
4	N	Valid	143	143	143	143
		Missing	0	0	0	0
	Mean		8.13	78.30	62.69	179.78
	Median		8.00	77.00	61.00	91.00
	Std. Deviation		2.57	13.68	11.60	338.65
	Minimum		2	51	37	14.60
	Maximum		11	125	114	3545.20
5	N	Valid	143	143	143	143
		Missing	0	0	0	0
	Mean		8.39	78.18	62.48	194.06
	Median		9.00	77.00	61.00	84.10
	Std. Deviation		2.50	13.78	11.02	376.41
	Minimum		2	51	37	14.60
	Maximum		11	125	114	3545.20
Pooled	N	Valid	143	143	143	143
		Missing	0	0	0	0
	Mean		8.26	78.23	62.61	186.34

Note. Predictors used for multiple data imputation were age, gender, income, body height, body weight, resting heart rate, NT-proBNP, presence of diseases such as atrial fibrillation, type 2 diabetes mellitus, hypertension, dyslipidemia and peripheral arterial disease, intake of medications such as anticoagulants,

smoking status, alcohol consumption, metabolic equivalents (MET), Mediterranean Diet Adherence Screener (MEDAS), Healthy Eating Index (HEI), diet change within past 12 months.

Table S7. Comparison of main variables pre- and post-case-control matching

	Pre-matching				Post-matching			
	Patients (n = 61)	Controls (n = 75)			Patients (n = 52)	Controls (n = 52)		
**Metric variables**	**M (SD)**	**M (SD)**	**t[a]**	**p**	**M (SD)**	**M (SD)**	**t[a]**	**p**
Body mass index, kg/m^2	26.2 (3.3)	24.0 (2.7)	4.29	< 0.001	26.4 (3.3)	24.2 (2.7)	3.66	< 0.001
Resting heart rate, bpm	66.0 (12.5)	60.1 (9.0)	3.15	0.002	64.9 (12.5)	60.2 (9.1)	2.14	0.035
MEDAS	2.5 (1.7)	3.6 (2.0)	-3.43	0.001	2.5 (1.7)	3.3 (1.6)	-2.33	0.022
HEI	50.1 (9.5)	56.8 (10.2)	-3.95	< 0.001	50.8 (9.5)	56.8 (10.3)	-3.06	0.003
Energy intake, kcal	2148.7 (554.5)	2188.1 (443.6)	-0.46	0.646	2186.1 (562.5)	2217.9 (433.6)	-0.32	0.747
Log NT-proBNP, pg/ml	2.2 (0.5)	1.8 (0.3)	5.98	< 0.001	2.3 (0.5)	1.8 (0.3)	6.44	< 0.001
Physical activity, log MET-h/day	3.4 (0.5)	3.3 (0.3)	0.66	0.511	3.4 (0.4)	3.3 (0.4)	1.40	0.166
**Nominal/ordinal variables**	**n (%)**	**n (%)**	**Chi^2 [b]**	**p**	**n (%)**	**n (%)**	**Chi^2 [b]**	**p**
No diet change past 12 months	47 (77.0%)	61 (81.3%)	0.38	0.670	40 (76.9%)	43 (82.7%)	0.54	0.626
Income categories			0.34	0.863			0.35	0.866
low (< 2500€)	10 (16.4%)	11 (14.7%)			9 (17.3%)	10 (19.2%)		
middle (2500€ < 5000€)	35 (57.4%)	41 (54.7%)			29 (55.8%)	26 (50.0%)		
high (> 5000€)	16 (26.2%)	23 (30.7%)			14 (26.9%)	16 (30.8%)		

Note. [a]t-Test. [b]Pearson's Chi Square test.

MEDAS, Mediterranean Diet Adherence Screener; HEI, Healthy Eating Index
